# Intranasal M2SR and BM2SR Vaccine Viruses Do Not Shed or Transmit in Ferrets

**DOI:** 10.3390/vaccines12111228

**Published:** 2024-10-29

**Authors:** Yasuko Hatta, Lindsay Hill-Batorski, Michael J. Moser, David Marshall, David A. Boltz, Landon Westfall, Renee Herber, Sally Sarawar, Pamuk Bilsel

**Affiliations:** 1 FluGen, Inc., Madison, WI 53711, USA; 2 IIT Research Institute, Chicago, IL 60616, USA; 3 The BioMedical Research Institute of Southern California, Oceanside, CA 92056, USA

**Keywords:** M2-deficient, shedding, live, influenza, M2, BM2, transmission, vaccine, single-replication, ferret, intranasal, reassortment

## Abstract

Background/Objectives: Live influenza vaccines are considered to stimulate better overall immune responses but are associated with safety concerns regarding shedding and the potential for transmission or reassortment with wild-type influenza viruses. Intranasal M2SR and BM2SR (M2- and BM2-deficient single replication), intranasal influenza viruses, have shown promise as broadly cross-reactive next-generation influenza vaccines. The replication deficiency, shedding, and transmissibility of M2SR/BM2SR viruses were evaluated in a ferret model. Methods: Wild-type influenza A and B control viruses replicated in upper respiratory organs and transmitted to both direct and aerosol contact ferrets, whereas M2SR and BM2SR influenza vaccine viruses were not detected in any tissues or in nasal washes after inoculation and were not recovered from any direct or aerosol contact ferrets. Mice were simultaneously infected with wild-type influenza A and M2SR viruses to assess reassortment potential. Sequence and PCR analyses of the genome recovered from individual virus plaques isolated from lung homogenates identified the origin of the segments as exclusively from the replicating wild-type virus. Results: These results indicate that M2SR and BM2SR influenza vaccine viruses are attenuated, do not shed or transmit, and have a low probability for reassortment after coinfection. Absence of shedding was further demonstrated in nasal swabs taken from subjects who were inoculated with H3N2 M2SR in a previously described Phase 1 clinical study. Conclusions: These results indicate that M2SR/BM2SR viruses have the potential to be used in a broader population range than current live influenza vaccines.

## 1. Introduction

Influenza infection is a leading cause of morbidity and mortality worldwide, with a substantial health burden. Vaccination is the most effective method for prevention and control of influenza; however, current licensed vaccines are modest in performance, with a recent systematic review of influenza vaccine effectiveness (VE) after the 2009/10 influenza pandemic indicating pooled VE of 49% (95% CI 45–54%) against vaccine matched and −9% (95% CI −28–8%) for antigenically dissimilar vaccines [1]. A majority of licensed vaccines are inactivated or recombinant HA-based, injected intramuscularly, and rely on serum antibody responses against the HA as the mechanism of action. These intramuscular vaccines do not prevent infection but may prevent serious illness or death from influenza.

Live vaccines, in general, are thought to induce broader, more complete immune responses that engage multiple effector systems [2]. There is currently one licensed live-attenuated influenza vaccine (LAIV), registered as FluMist in the US and Fluenz in Europe, that is administered intranasally, but its use is limited mainly to children. LAIV in the pivotal Phase 3 study demonstrated 93% efficacy against culture-confirmed influenza in children 15 to 71 months old, but since then, its real-world effectiveness has been similar to inactivated influenza vaccines (IIVs) [3,4]. LAIV is a replicating virus that sheds in children for 7–10 days and for 3 days in adults. It is indicated only in children 2 years and older due to increased wheezing events and hospitalizations in children younger than 2 years. LAIV is also not indicated in adults over 50 years old, due to poor efficacy in this age group. Due to the risk of transmission associated with live influenza vaccines, LAIV currently is not recommended for those with close contacts at high risk for complications from influenza, such as immunocompromised individuals and pregnant women [5].

The investigational M2SR and BM2SR influenza vaccine viruses do not express M2 and BM2 proteins that are essential for replication and infection, resulting in a single-cycle infection that does not produce an infectious progeny virus [6,7]. Replication for M2SR/BM2SR viruses requires external supplies of M2 or BM2 proteins from production cells. In non-production cells that express neither M2 nor BM2, M2SR and BM2SR viruses infect and express all functional influenza viral proteins except M2 or BM2, but they produce no infectious progeny virions. Because the M2SR and BM2SR viruses do not undergo multi-cycle replication, many of the concerns that exist for live-attenuated vaccines, e.g., reversion, reassortment, shedding, and transmission, are not applicable to M2SR and BM2SR vaccine viruses.

Here, we assessed the safety profiles of M2SR and BM2SR influenza vaccine viruses in animal models and humans. Attenuation, shedding, and transmission of vaccine viruses were evaluated in ferrets, with verification of absence of shedding in human subjects. We also evaluated the potential for reassortment of M2SR with wild-type influenza in a mouse model.

## 2. Materials and Methods

### 2.1. Virus

M2SR vaccine virus, M2SR-Bris10, possessing HA and NA of influenza A/Brisbane/10/2007 (H3N2)-like influenza A/Uruguay/716/2007 (H3N2), respectively; and BM2SR vaccine virus, named BM2SR-CO/06, possessing HA and NA of influenza B/Colorado/06/2017 (Victoria lineage), were generated and amplified as described previously [7,8]. Wild-type (WT) influenza A/California/07/2009 (H1N1pdm) (A/CA/07), A/Aichi/2/68 (H3N2) (A/Aichi/68), A/Brisbane/10/2007 (H3N2) (A/Bris/10), and B/Brisbane/60/2008 (Victoria lineage) (B/Bris/60) were amplified in MDCK cells. The viruses were titrated in M2-expressing MDCK (M2CK), BM2-expressing MDCK (BM2CK), or MDCK cells [9,10].

### 2.2. Ferrets

Influenza-free male and female ferrets, 4–6 months of age, were purchased from Triple F Farms (Gillett, PA, USA). All animal procedures were performed in IIT Research Institute’s animal Biosafety level-2 suite in accordance with the protocols approved by the animal care and use committee at IIT Research Institute. Ferrets were monitored for 3 days to establish baseline body temperature and body weights prior to study initiation. Temperature readings were recorded daily through a transponder (BioMedic data systems, Seaford, DE, USA) implanted subcutaneously in each ferret.

### 2.3. Attenuation of M2SR and BM2SR Influenza Vaccine Viruses

Groups male ferrets (n = 3) were anesthetized and inoculated intranasally with a single dose of 1 × 10^7^ tissue culture infectious dose (TCID_50_) of M2SR-Bris10, a single dose of 1 × 10^7^ TCID_50_ of WT influenza A/Bris/10, a single dose of 1.6 × 10^8^ TCID_50_ of BM2SR-CO/06, or a single dose of 2.5 × 10^7^ TCID_50_ of WT influenza B/Bris/60. Daily monitoring of ferrets for body weight, body temperature, clinical signs (dyspnea, sneezing, coughing, and rhinorrhea) inappetence, and level of activity were done. Ferret activity levels were evaluated based on a scoring system by Reuman et al. [11] as follows: 0, alert and playful; 1, alert but playful only when stimulated; 2, alert but not playful when stimulated; and 3, neither alert nor playful when stimulated. During the study, a daily relative inactivity index (RII) was calculated by using the mean score per each group of ferrets. All ferrets were euthanized three days after inoculation and necropsied. Tissue samples were collected from respiratory and major organs that included nasal turbinates, trachea, lungs, pancreas, kidneys, olfactory bulbs, brains, spleens, livers and small and large intestines. One section of each collected sample was preserved for histological evaluation using formalin fixation, and the remaining section was frozen at ≤−65 °C for virus titration.

### 2.4. Transmission Studies in Ferrets

Groups of 3 donor ferrets (females for M2SR-Bris10 and A/Bris/10 and males for BM2SR-CO/06 and B/Bris/60) were anesthetized and inoculated intranasally, as described above in Section 2.3., Each inoculated donor was placed in the same cage with 1 naïve ferret (direct contact) within a wire cage (dual housed) twenty-four hours post-inoculation. An additional ferret (aerosol contact) was placed a distance of 10–12 cm in a separate adjacent wire cage (single housed) within the transmission chamber. Ferret body weights, body temperatures, and clinical signs were measured daily for 14 days post-inoculation, along with mortality assessments. The inoculated donor ferrets were subject to nasal-wash collection on days 1, 3, 5, 7, 9, and 14. Nasal washes from all contact (direct and aerosol) ferrets were performed on days 2, 4, 6, 8, 10, and 14. Nasal washes were evaluated by TCID_50_ infectivity assay for presence of virus. 

### 2.5. qRT:PCR Analysis of Human Nasal Specimens

Nasal swabs were collected from subjects 18–49 years old into viral transport media (VTM) on days 0 (pre-dose), 1, 2, 3, and 7 and frozen at ≤−65 °C in a phase 1, blinded, placebo-controlled, randomized, dose-escalation study, with M2SR described previously (Clinical Trials Registration: NCT02822105) [12]. Briefly, healthy adults were randomized 3:1 for M2SR or placebo into four cohorts: 10^6^ TCID; 10^7^ TCID_50_; and 10^8^ TCID_50_ M2SR or placebo. M2SR-Bris10 and placebo (sterile physiological saline) were intranasally administered using the VaxINator™ Intranasal Mucosal Atomization Device (Teleflex, Morrisville, NC, USA). Vaccine virus shedding was evaluated by real-time quantitative reverse-transcription polymerase chain reaction (qRT-PCR) and a TCID_50_ infectivity assay in M2CK cells [10]. Briefly, thawed nasal swabs were split into two aliquots: one aliquot evaluated in TCID_50_ assay [13] and one aliquot used for RNA extraction by magnetic silica beads for TaqMan qRT:PCR designed to detect influenza A Matrix gene, as described previously [14].

### 2.6. In Vivo Co-Infection

Three female BALB/c mice, 7 weeks old (Envigo, now Inotiv, Indianapolis, IN, USA) were inoculated intranasally with a mixture of 1 × 10^6^ plaque-forming units (PFU) of M2SR-Bris10 vaccine virus and 0.25 × 10^6^ PFU of WT influenza A/CA/07 virus. Three days after inoculation, mice were euthanized, and lungs were harvested. All study protocols were approved by the FluGen Institutional Animal Care and Use Committees in accordance with the National Institutes of Health guidelines for the care and use of laboratory animals. Lungs were homogenized and then centrifuged at 300× *g*, and the supernatants were inoculated onto MDCK cells for plaque isolation of the viruses. The plaque-isolated viruses were further amplified in MDCK cells. Total RNA was extracted from each virus isolate and from the individual mouse-lung homogenates. Complementary DNAs (cDNAs) were synthesized by reverse transcription with SuperScript II (ThermoFisher Scientific, Waltham, MA, USA), using influenza A-specific primer Uni12: 5′-AGCAAAAGCAGG-3′. The resulting cDNA samples were PCR amplified using HA, NA, PB1, and M segment- and strain-specific primer pairs (Supplementary Table S1) with POWER SYBR qPCR reagent and the BioRad CFX96 qPCR instrument to detect amplified products. Data were analyzed using the dCT method, where the qPCR cycle threshold (CT) value obtained with the A/CA/07 PCR primer amplification reactions is subtracted from the CT value obtained with the M2SR-Bris10 virus PCR primer systems. The identity of remaining influenza segments (NP, NS, PA, and PB2) was determined by genome sequencing (Sanger method).

## 3. Results

### 3.1. Single-Replication M2SR and BM2SR Viruses Do Not Disseminate in Ferrets

Ferrets were intranasally inoculated with M2SR or BM2SR influenza vaccine viruses or their respective WT influenza A or influenza B viruses and monitored for clinical signs of infection and survival. All ferrets survived infection. The ferrets inoculated with M2SR or BM2SR viruses did not exhibit any clinical symptoms or decrease in activity levels (Table 1). Ferrets inoculated with WT A/Bris/10/2007 presented respiratory signs (sneezing) on days 2 and 3 post-inoculation, along with decreased activity levels (Table 1). Ferrets inoculated with WT B/Bris/60/2008 did not show any clinical symptoms.

**Figure 1:**
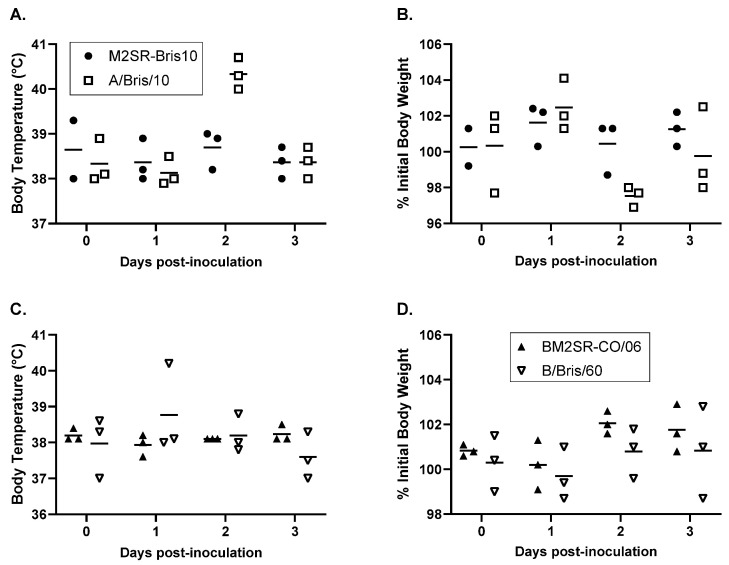
Body temperature and body-weight change after M2SR vaccine and wild-type influenza A virus inoculations (panels (**A**,**B**)) and after BM2SR and wild-type influenza B virus inoculations (panels (**C**,**D**)). Horizontal lines are group average of body temperature (°C) and of % initial body weight (vaccine group, filled circle or triangle; and wild-type virus group, open square or inverted triangle). Initial body weight was an average body weight of days 3–0 post-inoculation for individual ferret.

The M2SR or BM2SR vaccine viruses induced minimal-to-no weight loss in ferrets after intranasal inoculation (Figure 1B,D). In contrast, 2–3% weight loss was observed in the three ferrets inoculated with WT A/Bris/10/2007 on day 2 post-inoculation, and 3% weight loss was seen on day 1 post-inoculation in one ferret inoculated with WT B/Bris/60/2008 that did not return to starting weight until day 4 post-inoculation. The other two ferrets inoculated with WT B/Bris/60/2008 did not display any weight loss.

Elevated body temperatures of 40.3–40.7 °C were observed in ferrets inoculated with WT A/Bris/10/2007 on day 2 and in one of the ferrets inoculated with WT B/Bris/60/2008 on day 1 post-inoculation (Figure 1A,C). Body temperatures returned to normal range by day 3 post-inoculation. Body temperatures for M2SR- and BM2SR-inoculated ferrets remained in the normal range throughout the duration of the study.

Ferrets were necropsied on day 3 post-inoculation for evaluation of virus dissemination to the respiratory tract and major organs by virus titration and histopathology. No infectious virus was detected in any of the organs tested in ferrets inoculated with M2SR or BM2SR vaccine viruses, except for low-level virus detected in the pancreas of one ferret in the BM2SR group (Table 1). In contrast, the two wild-type viruses, A/Bris/10/2007 and B/Bris/60/2008, replicated mainly in the nasal turbinates, with mean log_10_ 5.43 and 3.60 TCID_50_, respectively. The WT A/Bris/10/2007 virus was not detected in any other organ, but WT B/Bris/60/2008 virus was detected in the trachea (1/3) of the infected ferrets (Table 1).

Tissues collected from ferrets inoculated with M2SR or BM2SR viruses displayed minimal histopathological changes associated with viral infection in the lungs or nasal turbinates (Table 2). In contrast, ferrets inoculated with WT A/Bris/10/2007 revealed microscopic changes in the nasal turbinates that included atrophy of the respiratory epithelium, infiltration of neutrophils, and edema (Table 2). Similarly, mild-to-moderate inflammatory cell infiltration was observed in the upper respiratory (nasal turbinate) and lungs in ferrets inoculated with WT B/Bris/60 (Table 2).

These data show that the M2SR and BM2SR influenza vaccine viruses do not induce clinical signs of infection, do not replicate in the respiratory tract, and do not disseminate to other tissues in ferrets.

### 3.2. M2SR and BM2SR Influenza Vaccine Viruses Do Not Shed or Transmit in Ferrets

Individually housed ferrets (donor ferrets) were inoculated with vaccine viruses M2SR or BM2SR and their respective wild-type viruses, A/Bris/10/2007 or B/Bris/60/2008. Twenty-four hours after inoculation, naïve ferrets were placed into the transmission chamber (direct contacts) or into an adjacent transmission chamber (aerosol contacts) to evaluate transmission. Nasal washes were collected every other day, starting on one day post-inoculation for donor ferrets and starting on two days post-inoculation for contact ferrets, and evaluated for virus. Ferrets infected with the vaccine viruses M2SR and BM2SR did not display any weight loss, fever, or clinical symptoms in contrast to those infected with wild-type A/Bris/10/2007 and B/Bris/60/2008 viruses that demonstrated sneezing, mild weight loss, and temperature rise after infection (Supplementary Figure S1 and Table S2).

**Figure 2:**
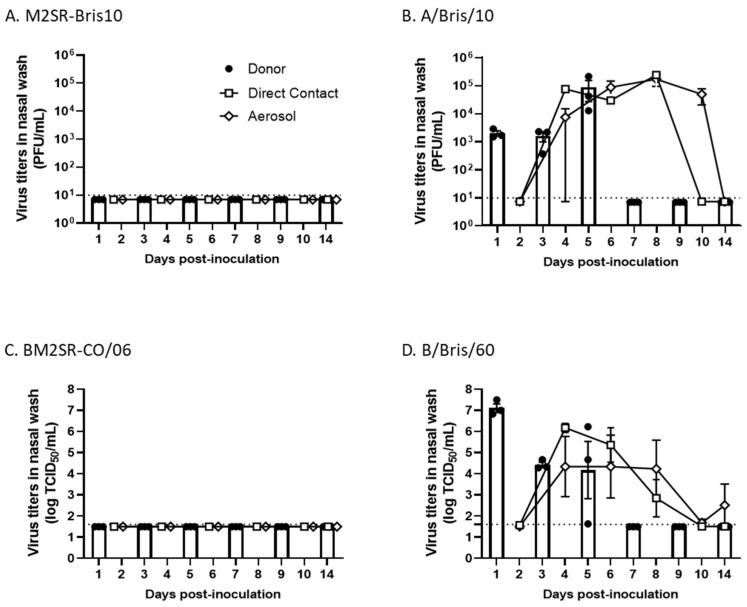
M2SR, BM2SR, and wild-type influenza A and B virus transmission. Nasal wash was harvested from donor (filled circle), direct contact (open square), and aerosol contact (open diamond) ferrets on the indicated days pot-inoculation. M2SR-Bris10 (panel (**A**)) and A/Bris/10 (panel (**B**)) were titrated in M2CK cells; BM2SR-CO/06 (panel (**C**)) was performed in BM2CK cells, and B/Bris/60 (panel (**D**)) was performed in MDCK cells. Individual nasal-wash virus titers (filled circle) and averages (bar) are plotted for donor ferrets. Average nasal wash virus titers for direct contact and for aerosol-contact ferrets are plotted as open squares and open diamonds, respectively. Error bars indicate standard error of the mean (SEM) calculated by Prism (GraphPad Software version 10.1.0 (316), San Diego, CA, USA). Dash lines indicate lowest detection limit (10 PFU/mL or 1.67 log_10_ TCID_50_/mL) for the assay. Individual ferret titer data are provided in Supplementary Table S3.

No virus was detected in the nasal washes from any of the donor or contact ferrets infected with M2SR or BM2SR viruses (Figure 2A,C). In contrast, shedding virus was detected on days 1, 3, and 5 post-inoculation in all donor ferrets infected with the wild-type A/Bris/10/2007 and B/Bris/60/2008 viruses (Figure 2B,D). Virus was detected in the nasal wash of all three direct-contact ferrets in the WT A/Bris/10/2007 group on days 4, 6, and 8 post-inoculation and in that of aerosol-contact ferrets on days 4 (1/3), 6 (3/3), 8 (3/3), and 10 (2/3) (Figure 2B). Virus was detected in the nasal wash of direct-contact ferrets in the WT B/Bris/60/2008 group on days 2 (1 of 3 ferrets), 4 (3 ferrets), 6 (3 ferrets), and 8 (2 of 3 ferrets) post-inoculation and that of aerosol-contact ferrets on day 4 (2/3), 6 (2/3), 8 (2/3), 10 (1/3), and 14 (1/3) (Figure 2D). None of the contact ferrets displayed any clinical signs of infection except for the direct-contact ferrets for wild-type influenza A H3N2 Brisbane/2007 virus (Supplementary Table S4).

These data demonstrate that the vaccine viruses M2SR and BM2SR are not shed from and do not transmit in ferrets.

### 3.3. M2SR Vaccine Virus Does Not Shed in Human Subjects

**Figure 3:**
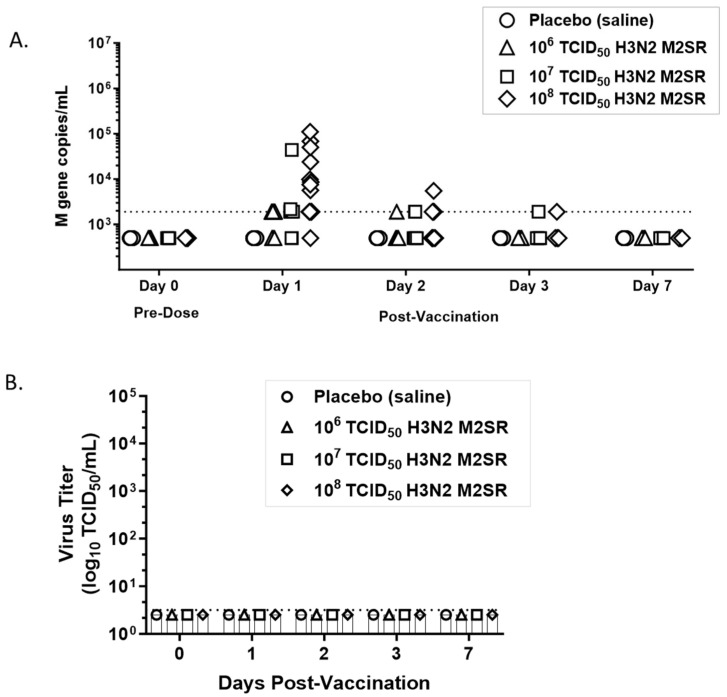
H3N2 M2SR vaccine virus shedding in human subjects. (**A**) qRT:PCR evaluation of nasal specimens from subjects vaccinated with H3N2 M2SR. (**B**) Infectivity evaluation by TCID_50_ assay in M2CK cells; n = 24 for each cohort except for 10^7^ dose, n = 23. Individual subject data are shown.

Nasal-swab specimens from subjects in a previously described first-in-human Phase 1 study [12] were evaluated for shedding of M2SR vaccine virus. Seventy-one samples representing M2SR-Bris10 (H3N2) at three dose levels, 10^6^ TCID_50_ (n = 24), 10^7^ TCID_50_ (n = 23), and 10^8^ TCID_50_ (n = 24), and twenty-four samples from placebo subjects were evaluated for the presence of influenza virus M gene by qRT:PCR. No samples were positive for influenza virus RNA at baseline before vaccination (Figure 3). After vaccination, the frequency and duration of detectable influenza RNA were dose-dependent. M gene RNA was detected in nasal swabs of 58% of 10^6^, 78% of 10^7^, and 96% of 10^8^ TCID_50_ M2SR vaccinated subjects on day 1 (Figure 3A). On day 2, only 8%, 13%, and 42% of 10^6^, 10^7^, and 10^8^ M2SR-treated subjects had detectable RNA, respectively. By day 3, 4% of 10^7^ and 8% of 10^8^ M2SR-treated subjects had detectable M gene RNA. No M gene RNA was detected in any of the M2SR-vaccinated subjects by day 7. Placebo subjects had no detectable M gene RNA at any timepoint (Figure 3A).

The remaining nasal specimens were evaluated for the presence of infectious M2SR virus by TCID_50_ assay in permissive M2CK cells. No vaccine virus was detected by TCID_50_ assay in any of the vaccinated subjects at any timepoint, indicating that M2SR does not shed in vaccinated subjects (Figure 3B).

In summary, the live, intranasal M2SR influenza vaccine did not display any evidence of shedding in human subjects.

### 3.4. Co-Infection of Mice with Wild-Type H1N1 Influenza Virus and H3N2 M2SR Does Not Generate Reassortant Viruses

To address the theoretical risk of reassortment between M2SR vaccine virus and wild-type influenza virus resulting in replication-competent reassortant virus, three BALB/c female mice were intranasally inoculated with a mixture of H3N2 M2SR-Bris10 (1 × 10^6^ PFU) and wild-type H1N1 influenza A/CA/07/2009 (0.25 × 10^6^ PFU). Mice showed clinical symptoms of infection (faster breathing, ruffled fur, and reduced activity levels) starting on day 2 after virus inoculation and lost ~15% of their initial body weight by day 3 post-inoculation, when they were euthanized to harvest their lungs (Table 3).

Virus titers greater than 10^7^ PFU per gram of lung tissue were detected in lung homogenates (Table 3). Our sequence analysis of RNA extracted from the lung homogenates detected trace amounts of the M2SR vaccine virus genome and copious amounts of wild-type H1N1 CA/07/2009, indicating that, as expected, the inoculated H3N2 M2SR did not amplify, while the WT H1N1 virus did. Single plaques from the lung homogenates were isolated and amplified in MDCK cells (non-permissive for M2SR), thus selecting for replication-competent viruses. The amplified plaques were then genotyped for the presence of genetic material derived from the M2SR vaccine virus. Seventy-six total plaque-purified viruses (46, 16, and 14 plaques from each of the three mice) were genotyped to determine the origin of the segments (Table 3).

No segments derived from the M2SR-Bris10 vaccine virus were detected during the analysis of any of the qPCR and DNA sequence data for the 76 total isolated plaques from the three lung homogenates (Table 3 and Supplementary Table S5). All segments for the replicating viruses were derived from the WT H1N1 virus. Despite the presence of both viruses in the mouse lung, no genetic exchange resulting in replication-competent reassortants was observed between the H3N2 M2SR vaccine and the wild-type H1N1 CA/07/2009 virus.

In summary, concomitant administration of M2SR and WT viruses in vivo does not result in replication-competent viruses containing M2SR segments.

## 4. Discussion

We report here that the intranasal, live single-replication vaccines M2SR and BM2SR are not shed or transmitted in ferrets. The absence of vaccine virus shedding was also demonstrated in nasal specimens from subjects who were administered H3N2 M2SR in the first-in-human clinical trial. These studies verify that the M2SR/BM2SR platforms, in which the essential M2/BM2 proteins are not expressed, result in non-transmissible viruses and thus make the M2SR platform ideal for use as live vaccines. A non-shedding live influenza vaccine eliminates many of the concerns associated with live influenza vaccines.

M2SR vaccines model natural (wild-type) virus infection, while incorporating the critical safety feature of a single round of virus replication after vaccine administration. With these properties, an M2SR seasonal vaccine may confer additional advantages over currently available influenza vaccines, including the live influenza vaccine. Currently, there is only one intranasal LAIV, marketed as FluMist or Fluenz, that has been licensed for use in healthy individuals aged 2 to 49 years old in the US and 2–18 years old in Europe. While LAIV is shed at high titers in children, it has shown a low probability of transmission, 0.58% (95% CI 0, 1.7), as reported in a day-care study designed to address the issue of transmission [15]. Of 460 cases of vaccine-strain transmission associated with LAIV that were reported to the Vaccine Adverse Event Reporting System between 2003 and 2005, secondary transmission accounted for only 22 (4.8%) of the events [16]. Despite the extremely low probability of transmission, concerns with live influenza vaccines exist regarding shedding and the potential for human-to-human transmission. Hence, LAIV is not indicated in certain settings, such as in those with close contacts who are immunocompromised [17]. Additionally, because of the potential for transmission [18], many pediatric practices appear hesitant to use LAIV in their offices due to the contraindications for pregnant women, contacts of immunocompromised individuals, and children with asthma, all of whom are common family members of pediatric patients.

The non-transmissible safety profile of the M2SR and BM2SR vaccines suggest that there would be no concerns regarding vaccine virus shedding or transmission. This could potentially expand the eligible populations that currently do not benefit from live influenza vaccines. H3N2 M2SR has been evaluated in over 700 subjects, ranging in age from 2 to 85 years old, and has been well-tolerated [12,19,20,21,22,23]. Along with this favorable safety profile, M2SR has demonstrated a multifaceted immunogenicity profile that distinguishes it from LAIV. H3N2 M2SR elicited ≥4-fold seroconversion in HAI titers in 71% of 18–49-year-old vaccine recipients [19] compared with 10% seroconversion in a similar population for FluMist [24]. Similarly, H3N2 M2SR elicited significant HAI, mucosal sIgA antibodies, and T-cell responses in older adults aged 65–85 years old [20], an age group for which FluMist is not indicated. Moreover, M2SR has demonstrated significant immune responses in seroprotected individuals who already have pre-existing influenza immunity [12,20]. In contrast, LAIV vaccination has been shown to be susceptible to pre-existing influenza immunity that inhibits the vaccine virus from replicating to induce an immune response and therefore is almost exclusively used in young children, not adults [25,26]. Studies in ferrets have recapitulated this difference in which M2SR induced immune responses in animals with flu immunity, while LAIV did not [8].

Shedding in subjects vaccinated with H3N2 M2SR virus was evaluated by nasal sampling over a 7-day time course designed to include the timepoints (within 4 days after infection) when influenza viral shedding is highest. We show that the M2SR vaccine virus does not persist and is not amplified within the subject airway. This is further supported by the observation that viral RNA (from either residual inoculum or infected epithelial cells) detectable on day 1 does not persist, indicating lack of amplification. No virus was detected in any subject at any timepoint, indicating that no active virus replication occurred in the nasal mucosa during the seven days following intranasal administration of the vaccine. In contrast, natural influenza virus infection demonstrates peak virus titers in nasal specimens on days 2–4, with virus titers ranging from 2.7 to 5.6 log_10_ copies/mL [27]. Similarly, FluMist, the only licensed live-attenuated influenza vaccine, sheds vaccine virus in recipients, and viral shedding decreases with increasing age [28,29]. None of the subjects in the M2SR study displayed any evidence of shedding virus.

With live influenza vaccines, there exists a theoretic possibility of reassortment within a vaccine recipient by co-infection with a circulating influenza virus that could result in genetic exchange of information with the wild-type virus and potential generation of replication competent M2SR vaccine viruses. Since influenza-infected cells become refractory to further infection by new influenza particles [30,31], this would most likely occur during a rare case of a co-incident wild-type infection at the time of inoculation with the single-replication M2SR vaccine. We showed that progeny virus from mice inoculated simultaneously with the M2SR Bris2007 H3N2 vaccine virus and a second easily distinguished wild-type H1N1 influenza virus did not result in replication-competent derivatives of M2SR. Despite the co-existence of both viruses in the mouse lung, no genetic exchange resulting in replication-competent reassortants was observed between the H3N2 M2SR vaccine and the wild-type H1N1 CA/07/2009 virus. These results suggest that coinfection of the same cell within an infected host is unlikely when one of the viruses is a non-spreading replication-deficient virus such as M2SR; that is, there appears to be a need for amplification of both viruses for coinfection of the same cell to occur. It is important to highlight that co-infection at the cellular level, not just co-infection of the subject, appears to be a necessary precursor for reassortment to occur in such a scenario. These results suggest that there is an extremely low probability that M2SR could regain replication competence when administered to an asymptomatic infected individual or when an M2SR-vaccinated individual encounters a live influenza virus.

Since M2SR undergoes only a single round of replication and is not shed after vaccine administration, many of the safety issues that restrict the use of LAIV are not anticipated to be concerns for M2SR, i.e., safety in younger children < 2 years old and in subjects with previous asthma or recurrent wheezing [32]. The single round of replication and lack of M2/BM2 expression by M2SR/BM2SR is expected to avoid the induction of pro-inflammatory cytokines and the inflammation that is associated with reactive airway disease. LAIV (like wild-type influenza) expresses M2 protein in infected cells and therefore activates NLRP3 inflammasomes, resulting in the production of the potent inflammatory cytokines IL-1β and IL-18 [33]. In the absence of expression of M2 protein, the dominant mechanism of inflammasome activation following influenza infection [33], M2SR is not expected to activate NLRP3 inflammasomes. The favorable safety profile of M2SR in adults and the mild histopathology observed after M2SR/BM2SR infection in ferrets suggests that these viruses do not induce a strong inflammatory response.

## 5. Conclusions

In summary, intranasal M2SR and BM2SR influenza vaccine viruses are not shed, nor do they transmit virus after intranasal inoculation. The single-cycle replication phenotype of these vaccine viruses renders reassortment with circulating influenza viruses unlikely. These safety characteristics suggest that M2SR/BM2SR viruses have the potential for use in populations highly vulnerable to influenza, such as infants aged ≥ 6 months, and immunocompromised or older adults.

## Figures and Tables

**Table 1:** Survival, clinical symptoms, and virus replication in ferrets after infection.

Virus/Vaccine n = 3/group	Clinical Signs			Virus Titers (Mean ± SD log_10_ PFU/g ^a^ or log_10_ TCID_50_/g ^b^) in Ferret Organs ^c^							
	Respiratory Signs (Day of Onset)	Loss of Appetite	Lethargy (RII)	Nasal Turbinate	Trachea	Lung	Pancreas	Brain	Olfactory Bulb	Small Intestine	Large Intestine
M2SR-Bris10	0	0	0	<1.0 ^a^	<1.0	<1.0	<3.0 ^d^	<1.0	<1.0	ND ^e^	ND
WT A/Bris/10	2	0	0.67	5.43 ± 0.26	<1.0	<1.0	<2.0 ^f^	<1.0	<1.0	ND	ND
BM2SR-CO/06	0	0	0	<1.5 ^b^	<1.5	<1.5	2.50, 2.75	<1.5	<1.5	<1.5	<1.5
WT B/Bris/60	0	0	0	3.50 ± 0.90	2.75	<1.5	<1.5	<1.5	<1.5	<1.5	<1.5

RII, relative inactivity index. ^a^ M2SR-Bris10 and A/Bris/10 virus titers were measured in M2CK cells by plaque assay, detection limit, 1.00 log_10_ PFU/mL; ^b^ BM2SR-CO/06 and B/Bris/60 virus titers were measured byTCID_50_ in BM2CK and MDCK cells, respectively; detection limit 1.50 log_10_ TCID_50_/mL. ^c^ When virus was not recovered from all three ferrets, individual titers were recorded. No virus was detected in kidney, liver, and spleen. ^d^ Below detection limit, 3.00 log_10_ PFU/mL, cell monolayer was damaged by homogenate, and plaques were not countable in the first 2 dilutions. ^e^ ND, not determined. ^f^ Below detection limit, 2.00 log_10_ PFU/mL, cell monolayer was damaged by homogenate, and plaques were not countable in the first dilution.

**Table 2:** Histopathological findings in ferret tissues after M2SR, BM2SR, or wild-type influenza A or B infection.

Tissue	Histopathological Observations	M2SR Bris10 n = 3	WT A/Bris/10/2007 n = 3	BM2SR CO/06 n = 3	WT B/Bris/60/2008 n = 3
Nasal turbinate	Within normal limits	0, 0, 0		0, 0	
	Atrophy, respiratory epithelium		3, 3, 3		
	Infiltration, neutrophilic		2, 2, 3		
	Edema		1, 2, 3		
	Edema, lumen			1 ^b^	1, 1, 2
	Infiltrate, mixed cell, turbinate				2, 2
	Edema, turbinate				2
	Infiltrate, mixed cell, lumen			1 ^b^	2, 3, 3
	Squamous metaplasia, turbinate				2, 2
Trachea	Within normal limits	0, 0, 0	0, 0, 0	0, 0, 0	0, 0, 0
Lung	Within normal limits	0, 0	0, 0	0	
	Perivascular/peribronchiolar mononuclear cell infiltration		1	1, 1	1, 1, 1
	Bronchiole syncytia	1	1		
	Alveolar mixed cell infiltrates			2	2, 2, 3
	Hyperplasia, bronchiolar				1, 2, 2
Pancreas	Within normal limits	0, 0, 0	0, 0, 0	0, 0, 0	0, 0, 0
Brain	Within normal limits	0, 0, 0	0, 0, 0	0, 0, 0	0, 0, 0
Olfactory bulb	Within normal limits	0, 0, 0	0, 0, 0	0, 0, 0	0, 0, 0
Intestine, small	Within normal limits	0, 0, 0	0, 0, 0	0, 0, 0	0, 0, 0
Intestine, large	Within normal limits	0, 0, 0	0, 0 ^a^	0, 0, 0	0, 0, 0
Kidney	Within normal limits	0	0, 0	0, 0, 0	0
	Tubular cyst	1			1
	Mineralization	1	1		1
Liver	Within normal limits				
	Glycogen accumulation/vacuolation, hepatocellular	1, 2, 2	1, 2, 2	2, 2, 3	1, 1, 2
	Mononuclear cell infiltrates	1, 1, 2	1, 1, 2	1, 2, 2	1, 1, 1
Spleen	Within normal limits	0, 0, 0	0, 0		
	Increased hematopoiesis			2, 2, 2	2, 2, 2
	Congestion		1		
	Depletion; hematopoietic		1		

Scoring: 0 = within normal limits; 1 = minimal; 2 = mild; 3 = moderate; 4 = marked; 5 = severe. ^a^ Large intestine of one ferret was not examined; it did not present on slide. ^b^ Turbinates mechanically destroyed bilaterally; microscopic exam was compromised.

**Table 3:** M2SR and wild-type virus co-infection in mice.

Mouse Number	Weight Loss ^a^	Virus Titer in Lungs ^a^ (PFU/g Tissue)	Amplified Plaques/ Total Plaques	Plaques Genotyped, n	Genotype Characterization (Source, Number) H1N1 WT:H3N2 M2SR							
					HA	NA	PB2	PB1	PA	NP	M	NS
1	15.8%	1.54 × 10^7^	82/86	46	45:0 ^b^	46:0	46:0	45:0 ^b^	45:0 ^b^	46:0	46:0	46:0
2	14.9%	2.63 × 10^7^	20/20	16	16:0	16:0	16:0	16:0	16:0	16:0	16:0	16:0
3	13.6%	2.63 × 10^7^	31/32	14	14:0	14:0	14:0	14:0	14:0	14:0	14:0	14:0

^a^ Day 3 post-inoculation. ^b^ One plaque indicated a mixed genotype for these genes.

## Data Availability

The data presented in this study are available upon request from the corresponding author.

## Supplementary Materials

The following supporting information can be downloaded at https://www.mdpi.com/article/10.3390/vaccines12111228/s1, Table S1: Primer pairs for influenza A strain and segment-specific qPCR and sequencing; Figure S1: Temperature and weight change in donor and contact ferrets; Table S2: Individual ferret body weight and temperature data; Table S3: Individual ferret virus titers for donor and contact ferrets; Table S4: Clinical signs in inoculated donor and contact ferrets; Table S5: Genotype of plaque-purified virus strains from co-infection in mouse lung.
