# Supplementary material for: Intranasal M2SR and BM2SR Vaccine Viruses Do Not Shed or Transmit in Ferrets
Source: Vaccines (Basel). 2024 Oct 29;12(11):1228. doi: 10.3390/vaccines12111228 (PMC11598709; doi:10.3390/vaccines12111228)
Supplement: Supplementary file 1 [file vaccines-12-01228-s001.zip › Supplementary_Data_18OCT2024clean.pdf]

Supplementary Table 1. Primer Pairs for Influenza A Strain and Segment-Specific qPCR and sequencing

Target strain and segment	qPCR Primer name	Sequence 5'-3'
M2SR-Bris10, HA	FOR: FluA HA 1 to 15	AGCAAAAGCAGGGGA
	REV: Bris10 HA 81 to 63	TTTGAGCGAAAACCAGACA
A/CA/07, HA	FOR: FluA HA 1 to 15	AGCAAAAGCAGGGGA
	REV: CA07 HA 86 to 69	GACAGGAGTCTGCATTTGCGGTTGC
M2SR-Bris10, M	FOR: PR8 M2SR 768 to 787	TGCAACGGTTCAAGTGATTA
	REV: FluA M 916 to 897	ACTCCTTCCGTAGAAGGCCC
A/CA/07, M	FOR: CA07 M2 797 to 815	ATTGGGATCTTGCACCTGA
	REV: FluA M 916 to 897	ACTCCTTCCGTAGAAGGCCC
M2SR-Bris10, NA	FOR: FluA NA 1 to 15	AGCAAAAGCAGGAGT
	REV: Bris10 NA 92 to 69	GCATGAAGAAGCATATTGTGGA
A/CA/07, NA	FOR: FluA NA 1 to 15	AGCAAAAGCAGGAGT
	REV: CA07 NA 100 to 76	CCAATTTGTAATATTAAGTTAGCCA
M2SR-Bris10, PB1	PR8 PB1 351 to 369	AACGATGGAGGTTGTTCAG
	PR8 PB1 513 to 494	GAGCCTTCCAGACTCATTGG
A/CA/07, PB1	CA07 PB1 351 to 369	AACAATGGAAGTTGTTCAA
	CA07 PB1 513 to 494	TAGCCTTCCTGACTCATTAG
**Target segment**	**PCR Primer Pair**	**Sequence 5'-3'**
NS	FOR: FluA HA 1 to 14	CGAAGTTGGGGGGGAGCAAAAGCAGGGG
	REV: FluA NS 890 to 871	GGCCGCCGGGTTATTAGTAGAAACAAGGGTGTTTT
M, PB2, and PA	FOR: FluA M 1 to 15	CACACACGTCTCCGGGAGCAAAAGCAGGTAG
	REV: FluA M 1027 to	CACACACGTCTCCTATTAGTAGAAACAAGGTAGTTTTT
NP	FOR: FluA NP 1 to 26	CACACACGTCTCCGGGAGCAAAAGCAGGGTAGATAATCACTC
	REV: FluA NP 1565 to 1546	CACACACGTCTCCTATTAGTAGAAACAAGGGTATTTTT
**Target segment**	**Sequencing Primer**	**Sequence 5'-3'**
PB2	PB2 320 to 335	GGTGGAATAGGAATGG
PA	PA 1701 to 1719	GCCCATGTTCTTGTATGTG
NP	NP 338 to 357	GGAGAGTAAACGGAAAGTGG
M	M 211 to 228	TGTGTTCACGCTCACCGT
NS	NS 606 to 586	CTCGAACTGTGTTATCATTCC

Supplementary Figure 1. Body temperature and body weight change in donor and contact ferrets after M2SR, BM2SR, and wild-type influenza A and B virus inoculation.

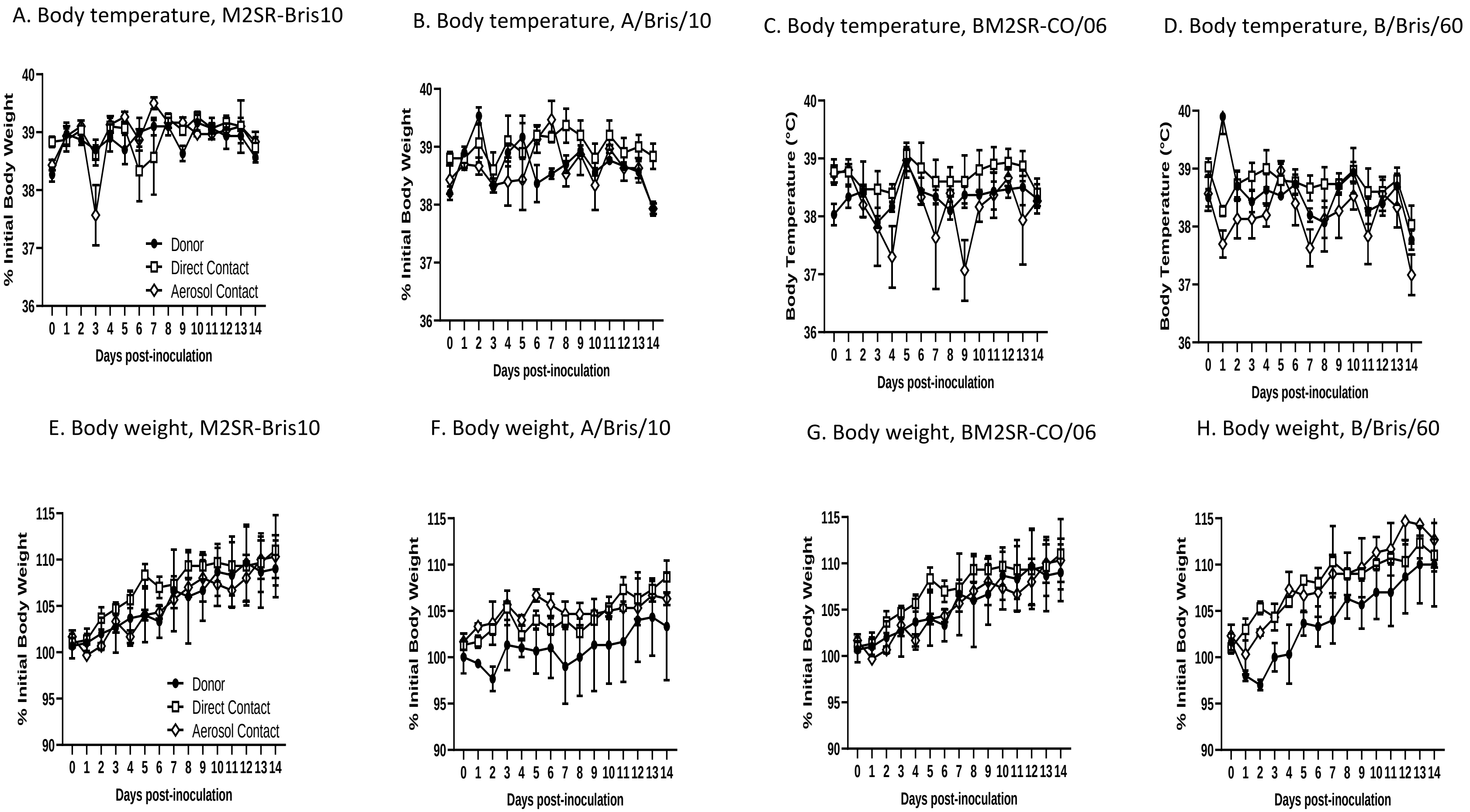

Body temperature (panels A-D) and body weight (panels E-H) were measured daily from donor, direct contact, and aerosol contact ferrets. Dots are group average of body temperature (°C) and of % initial body weight (donor ferrets: filled circle, direct contact ferrets: open square, and aerosol contact ferrets: open diamond). Error bars indicate standard error of the mean (SEM) calculated by Prism (GraphPad Software, San Diego, CA). Initial body weight was an average body weight of days -3–0 pre-inoculation for individual ferret. Individual ferret data follows in Supplementary Table S2.

Supplementary Table S2. Individual Ferret Body Weight and Temperature Data for Donor and Contact Ferrets

M2SR Body Weight

Day	Donor 1	Donor 2	Donor 3	Direct 1	Direct 2	Direct 3	Aerosol 1	Aerosol 2	Aerosol 3
0	102	98	102	102	101	100	103	101	101
1	99	102	102	99	102	103	99	100	100
2	99	105	102	102	103	106	101	101	100
3	99	108	101	104	106	104	101	105	104
4	98	108	105	107	106	104	101	101	103
5	99	109	104	109	110	106	103	105	104
6	100	106	104	109	105	107	103	105	105
7	100	115	105	107	109	106	104	106	107
8	100	116	102	109	112	107	105	108	108
9	102	113	105	109	112	107	103	110	111
10	104	113	109	110	113	106	103	108	111
11	103	115	107	112	113	103	104	106	110
12	102	116	111	112	115	101	105	106	113
13	103	116	107	110	115	104	106	111	113
14	103	113	111	112	117	104	106	111	114

WT Bris10 Body Weight

Day	Donor 1	Donor 2	Donor 3	Direct 1	Direct 2	Direct 3	Aerosol 1	Aerosol 2	Aerosol 3
0	103	100	97	102	99	103	100	103	102
1	100	99	99	103	101	101	104	103	103
2	99	95	99	102	103	104	100	108	103
3	103	96	105	103	104	109	106	106	105
4	103	100	100	104	103	100	104	103	105
5	104	96	102	103	106	103	106	108	106
6	106	95	102	102	103	104	104	105	108
7	104	91	102	104	104	104	106	106	102
8	106	92	102	102	103	103	104	103	107
9	109	92	103	104	104	104	104	105	105
10	106	93	105	103	106	107	104	105	106
11	106	93	106	106	106	110	106	105	105
12	109	95	108	104	103	112	107	103	106
13	109	96	108	107	106	109	106	108	106
14	107	92	111	108	106	112	107	105	107

M2SR Body Temperature

Day	Donor 1	Donor 2	Donor 3	Direct 1	Direct 2	Direct 3	Aerosol 1	Aerosol 2	Aerosol 3
0	38.2	38.5	38.1	39	38.7	38.8	38.6	38.4	38.3
1	39.3	38.6	39	38.9	39.2	38.5	38.6	39.1	39.1
2	38.7	39	38.9	39.2	39.2	38.7		39.2	39
3	38.7	38.4	39	38.9	38.6	38.3	38.6	37.1	37
4	38.5	39.3	38.9	39	39.4	38.9	38.9	39.4	39.1
5	38.5	39.2	38.4	39	39.2	39	39.3	39.4	39.1
6	38.5	39.2	39.3	38.7	37.3	39	39.1	39	38.5
7	38.8	39.3	39.2	39.4	37.3	39	39.7	39.4	39.4
8	38.8	39.3	39.2	39.2	39.1	39.4	39.3	39.2	39
9	38.5	38.5	38.9	38.9	39.1	39.1	39	39.2	39.3
10	38.9	39.3	39.3	39.3	39.4	39.1	39	39	38.9
11	38.7	39.2	39.3	38.9	39.2	39.1	39.1	39	38.8
12	38.5	39.2	39.1	39.4	39	39.1	39.2	39	38.9
13	39	38.7	39.1	39.3	38.8	39.2	39.6	39.5	38.2
14	38.4	38.6	38.7	38.6	38.5	39.1	39.1	38.5	38.9

WT Bris10 Body Tempearture

Day	Donor 1	Donor 2	Donor 3	Direct 1	Direct 2	Direct 3	Aerosol 1	Aerosol 2	Aerosol 3
0	38.4	38	38.2	39	38.8	38.6	38.1	38.3	38.9
1	38.9	38.9	38.9	39.2	38.6	38.6	38.8	38.6	38.7
2	39.8	39.5	39.3	39.3	39.5	38.4	38.4	38.9	38.7
3	38.3	38.4	38.2	39.2	38.2	38.4	38.4	38.1	38.5
4	38.8	39.2	38.7	39.2	39.8	38.3	37.6	39	38.6
5	38.7	39.9	38.9	39.9	38.6	38.2	37.4	39.1	38.8
6	37.8	38.9	38.4	39.1	39.5	39	39.6	38.9	38.9
7	38.4	38.5	38.7	39.2	39.3	39	39.3	39	40.1
8	38.6	39	38.5	39.8	39.5	38.8	38.1	38.7	38.8
9	39.1	38.9	38.8	39.4	39.5	38.7	38.2	39	39.4
10	38.7	38.5	38.5	39.3	38.6	38.5	37.5	38.6	38.9
11	38.7	38.8	38.8	39.5	39.4	38.7	38.9	38.9	39.1
12	38.6	38.6	38.8	39.1	39.2	38.4	38.2	38.9	38.8
13	38.8	38.2	38.7	39.3	39.1	38.6	38.3	38.9	38.7
14	38	37.7	38.1	39.1	39	38.4	37.8	38.1	37.9

BM2SR Body Weight

Day	Donor 1	Donor 2	Donor 3	Direct 1	Direct 2	Direct 3	Aerosol 1	Aerosol 2	Aerosol 3
0	102	98	102	102	101	100	103	101	101
1	99	102	102	99	102	103	99	100	100
2	99	105	102	102	103	106	101	101	100
3	99	108	101	104	106	104	101	105	104
4	98	108	105	107	106	104	101	101	103
5	99	109	104	109	110	106	103	105	104
6	100	106	104	109	105	107	103	105	105
7	100	115	105	107	109	106	104	106	107
8	100	116	102	109	112	107	105	108	108
9	102	113	105	109	112	107	103	110	111
10	104	113	109	110	113	106	103	108	111
11	103	115	107	112	113	103	104	106	110
12	102	116	111	112	115	101	105	106	113
13	103	116	107	110	115	104	106	111	113
14	103	113	111	112	117	104	106	111	114

B/Bris60 WT Body Weight

Day	Donor 1	Donor 2	Donor 3	Direct 1	Direct 2	Direct 3	Aerosol 1	Aerosol 2	Aerosol 3
0	102	100	103	101	100	102	100	103	104
1	99	98	97	103	101	105	102	96	103
2	97	96	98	106	106	104	103	103	102
3	102	97	101	105	104	104	107	102	104
4	103	94	104	106	105	107	111	106	105
5	105	101	105	108	108	109	110	104	106
6	106	99	105	107	108	109	111	102	108
7	107	99	106	107	106	118	113	106	108
8	109	102	108	110	108	109	113	105	109
9	106	101	110	110	108	109	115	104	110
10	112	102	107	110	109	111	113	108	113
11	109	100	112	110	112	110	114	106	115
12	114	101	111	114	108	109	118	111	115
13	116	102	112	114	109	114	116	112	115
14	114	101	115	114	108	111	117	107	114

BM2SR Body Temperature

Day	Donor 1	Donor 2	Donor 3	Direct 1	Direct 2	Direct 3	Aerosol 1	Aerosol 2	Aerosol 3
0	37.8	37.9	38.4	39.2	38.5	38.6	39	38.5	38.7
1	38.7	38.1	38.2	39.2	38.6	38.5	39	38.7	38.7
2	38.3	38.6	38.4	39.4	37.8	38.2	37.9	38.6	38.1
3	38	38	37.7	39.1	38.2	38.1	38.4	38.5	36.5
4	38	38.3	38.2	38.7	38.3	38.2	36.5	38.3	37.1
5	38.9	38.5	39.4	39.4	39.1	38.7	39.2	39.1	38.6
6	38.8	38.5	38	39.7	38.5	38.3	38.1	38.5	38.4
7	38.5	38.4	38.1	39.2	37.9	38.7	38.8	38.2	35.9
8	38.2	38.3	37.8	39.1	38.3	38.4	38.3	38.4	38.5
9	38.1	38.6	38.4	39.5	38.2	38.1	38	37	36.2
10	38.3	38.5	38.3	39.4	38.2	38.8	38.2	38.6	37.7
11	38.7	38.8	37.8	39.5	38.6	38.6	38.6	38.9	37.6
12	38.5	38.7	38.2	39.3	38.5	39	38.9	38.7	38.4
13	39.1	38.1	38.3	39.3	38.9	38.4	38.7	38.7	36.4
14	38.8	38.1	38	38.9	38.2	38.1	38.3	38.4	38.1

B/Bris60 WT Body Temperature

Day	Donor 1	Donor 2	Donor 3	Direct 1	Direct 2	Direct 3	Aerosol 1	Aerosol 2	Aerosol 3
0	38.4	38.3	38.8	39.3	38.8	39	38.1	39.1	38.5
1	39.7	39.5	40.5	38.4	38.3	38.1	37.7	38.1	37.3
2	38.4	38.5	39.2	39.2	38.5	38.5	38.8	37.8	37.8
3	38.3	38.2	38.8	39.3	38.5	38.8	37.8	38.8	37.8
4	38.1	38.8	39	39.6	38.5	38.9	38.4	38.4	37.8
5	38.5	38.6	38.5	39.3	38.3	38.8	38.8	39.3	38.8
6	38.4	38.9	38.9	39.2	38.5	38.6	37.8	39.1	38.3
7	38.2	38	38.4	39.1	38.4	38.5	37	38	37.9
8	38.1	37.6	38.5	39.2	38.2	38.8	37.3	39.2	37.9
9	38.5	38.9	38.7	39.1	38.5	38.6	37.5	39.1	38.2
10	38.7	38.8	39.3	39.5	38.2	39.2	38.2	39	38.4
11	38	38.1	38.7	39.4	38.1	38.3	36.9	38.5	38.1
12	38	38.5	38.7	39	38.3	38.5	38.7	39	38
13	38.1	38.8	39.2	39	38.6	38.8	37.7	38.9	38.4
14	37.6	37.6	38.1	38.5	37.4	38.2	36.5	37.7	37.3

Supplementary Table S3. Individual Ferret Virus Titers for Donor and Contact Ferrets

		Nasal Wash Viral Titers (pfu/mL)										
		Days Post-Infection of Donor Ferret										
Dose Group	Animal	1	2	3	4	5	6	7	8	9	10	14
	**Donor 1**	<10		<10		<10		<10		<10		<10
	**Donor 2**	<10		<10		<10		<10		<10		<10
	**Donor 3**	<10		<10		<10		<10		<10		<10
	**Contact 1**		<10		<10		<10		<10		<10	<10
M2SR-Bris10	**Contact 2**		<10		<10		<10		<10		<10	<10
	**Contact 3**		<10		<10		<10		<10		<10	<10
	**Aerosol 1**		<10		<10		<10		<10		<10	<10
	**Aerosol 2**		<10		<10		<10		<10		<10	<10
	**Aerosol 3**		<10		<10		<10		<10		<10	<10
	**Donor 1**	1700		2200		43000		<10		<10		<10
	**Donor 2**	1500		2300		215000		<10		<10		<10
	**Donor 3**	2900		370		13000		<10		<10		<10
	**Contact 1**		<10		6000		63000		170000		<10	<10
WT A/Bris/10	**Contact 2**		<10		180000		20500		525000		<10	<10
	**Contact 3**		<10		33500		2050		300		<10	<10
	**Aerosol 1**		<10		<10		195000		37000		47000	<10
	**Aerosol 2**		<10		21500		55500		165000		<10	<10
	**Aerosol 3**		<10		<10		2200		295000		95000	<10

		Nasal Wash Viral Titers (log TCID50/mL)										
		Days Post-Infection of Donor Ferret										
Dose Group	Animal	1	2	3	4	5	6	7	8	9	10	14
	**Donor 1**	1.50		1.50		1.50		1.50		1.50		1.50
	**Donor 2**	1.50		1.50		1.50		1.50		1.50		1.50
	**Donor 3**	1.50		1.50		1.50		1.50		1.50		1.50
	**Contact 1**		1.50		1.50		1.50		1.50		1.50	1.50
BM2SR-CO/06	**Contact 2**		1.50		1.50		1.50		1.50		1.50	1.50
	**Contact 3**		1.50		1.50		1.50		1.50		1.50	1.50
	**Aerosol 1**		1.50		1.50		1.50		1.50		1.50	1.50
	**Aerosol 2**		1.50		1.50		1.50		1.50		1.50	1.50
	**Aerosol 3**		1.50		1.50		1.50		1.50		1.50	1.50
	**Donor 1**	7.50		4.33		1.63		1.50		1.50		1.5
	**Donor 2**	6.83		4.28		6.23		1.50		1.50		1.5
	**Donor 3**	7.00		4.67		4.67		1.50		1.50		1.50
	**Contact 1**		1.67		5.77		4.00		4.50		1.50	1.50
WT B/Bris/60	**Contact 2**		1.50		6.50		6.83		1.50		1.50	1.50
	**Contact 3**		1.50		6.23		5.23		2.50		1.50	1.50
	**Aerosol 1**		1.50		6.00		5.00		5.50		1.50	1.50
	**Aerosol 2**		1.50		5.50		6.50		5.67		1.50	1.50
	**Aerosol 3**		1.50		1.50		1.50		1.50		2.00	4.50

Supplementary Table S4. Clinical signs in inoculated donor ferrets and contact ferrets from transmission studies.

Inoculum vaccine/virus	Group	Survivors/ Total number	Clinical signs[a]		
			Respiratory signs (observed day of onset)	Loss of Appetite	Lethargy (RII)[b]
M2SR-Bris10	Donors	3/3	0/3	0/3	0
	Direct contact	3/3	0/3	0/3	0
	Aerosol contact	3/3	0/3	0/3	0
WT A/Bris/10	Donors	3/3	2/3 (6, 8)	0/3	0
	Direct contact	3/3	3/3 (8, 8, 8)	0/3	0
	Aerosol contact	3/3	0/3	0/3	0
BM2SR-CO/06	Donors	3/3	0/3	0/3	0
	Direct contact	3/3	0/3	0/3	0
	Aerosol contact	3/3	0/3	0/3	0
WT B/Bris/60	Donors	3/3	0/3	0/3	0
	Direct contact	3/3	0/3	0/3	0
	Aerosol contact	3/3	0/3	0/3	0

[a] Clinical signs were observed for 14 days after virus inoculation. Except for lethargy, findings for clinical signs are given as the number of ferrets with sign/total number. Respiratory signs were sneezing, day of onset for each ferret in parentheses.

[b] Determined twice daily for 14 days of observation based on the scoring system and was calculated as the mean score per group of ferrets per observation (day) over the 14-day period. The relative inactivity index (RII) before inoculation was 0.

Supplemental Table S5. Genotype of Plaque-Purified Virus Strains from Co-Infection in Mouse Lung

Method:	qPCR	qPCR	Sequence	qPCR	Sequence	Sequence	Sequence	Sequence
Segment:	4	6	1	2	3	5	7	8
Gene:	HA	NA	PB2	PB1	PA	NP	M1/M2	NS
1-Lung	H1	N1	CA07	CA07	CA07	CA07	CA07	CA07
2-Lung	H1	N1	CA07	CA07	CA07	CA07	CA07	CA07
3-Lung	H1	N1	CA07	CA07	CA07	CA07	CA07	CA07
1-1	H1	N1	CA07	CA07	CA07	CA07	CA07	CA07
1-2	H1	N1	CA07	CA07	CA07	CA07	CA07	CA07
1-3	H1	N1	CA07	CA07	CA07	CA07	CA07	CA07
1-4	H1	N1	CA07	CA07	CA07	CA07	CA07	CA07
1-5	H1	N1	CA07	CA07	CA07	CA07	CA07	CA07
1-6	H1	N1	CA07	CA07	CA07	CA07	CA07	CA07
1-7	H1	N1	CA07	CA07	CA07	CA07	CA07	CA07
1-8	H1	N1	CA07	CA07	CA07	CA07	CA07	CA07
1-9	H1	N1	G237G<br>CA07<br>S279F	CA07	CA07	CA07	CA07	CA07
1-10	H1	N1	CA07	CA07	CA07	CA07	CA07	CA07
1-11	H1	N1	CA07	CA07	CA07	CA07	CA07	CA07
1-12	H1	N1	CA07	CA07	CA07	CA07	CA07	CA07
1-13	H1	N1	CA07	CA07	CA07	CA07	CA07	CA07
1-14	FAIL	FAIL	FAIL	FAIL	FAIL	FAIL	FAIL	P164H<br>CA07
1-15	H1	N1	CA07	CA07	CA07	CA07	CA07	CA07
1-16	H1	N1	CA07	CA07	CA07	CA07	CA07	CA07
1-17	H1	N1	CA07	CA07	CA07	CA07	CA07	CA07
1-18	H1	N1	CA07	CA07	CA07	CA07	CA07	CA07
1-19	H1	N1	CA07	CA07	CA07	CA07	CA07	CA07
1-20	H1	N1	CA07	CA07	CA07	CA07	CA07	CA07
1-21	H1	N1	CA07	CA07	CA07	CA07	CA07	CA07
1-22	H1	N1	CA07	CA07	CA07	CA07	CA07	CA07
1-23	H1	N1	CA07	CA07	CA07	CA07	CA07	CA07
1-24	H1	N1	CA07	CA07	CA07	CA07	CA07	CA07
1-25	H1	N1	CA07	CA07	CA07	CA07	CA07	CA07
1-26	H1	N1	CA07	CA07	CA07	CA07	CA07	CA07
1-27	H1	N1	CA07	CA07	CA07	CA07	CA07	CA07
1-28	H1	N1	CA07	CA07	CA07	CA07	CA07	CA07
1-29	H1	N1	CA07	CA07	CA07	CA07	CA07	CA07
1-30	H1	N1	CA07	CA07	CA07	CA07	CA07	CA07
1-31	H1	N1	CA07	CA07	CA07	CA07	CA07	CA07
1-32	H1	N1	CA07	CA07	CA07	CA07	CA07	CA07
1-33	H1	N1	CA07	CA07	CA07	CA07	CA07	CA07
1-34	H1	N1	CA07	CA07	CA07	CA07	CA07	CA07
1-35	H1	N1	CA07	CA07	CA07	CA07	CA07	CA07
1-36	H1	N1	CA07	CA07	CA07	CA07	CA07	CA07

1-37	H1	N1	CA07	CA07	CA07	CA07	CA07	CA07
1-38	H1	N1	CA07	CA07	CA07	CA07	CA07	CA07
1-39	H1	N1	CA07	CA07	CA07	CA07	CA07	CA07
1-40	MIX	N1	CA07	CA07	CA07	CA07	CA07	CA07
1-41	H1	N1	CA07	CA07	CA07	CA07	CA07	CA07
1-42	H1	N1	CA07	CA07	CA07	CA07	CA07	CA07
								P167S
1-43	H1	N1	CA07	CA07	CA07	CA07	CA07	CA07
1-44	FAIL	FAIL	FAIL	MIX	MIX	FAIL	CA07	CA07
1-45	H1	N1	CA07	CA07	CA07	CA07	CA07	CA07
1-46	H1	N1	CA07	CA07	CA07	CA07	CA07	CA07
1-47	H1	N1	CA07	CA07	CA07	CA07	CA07	CA07
1-48	H1	N1	CA07	CA07	CA07	CA07	CA07	CA07
			S279F					
2-1	H1	N1	CA07	CA07	CA07	CA07	CA07	CA07
2-2	H1	N1	CA07	CA07	CA07	CA07	CA07	CA07
2-3	H1	N1	CA07	CA07	CA07	CA07	CA07	CA07
2-4	H1	N1	CA07	CA07	CA07	CA07	CA07	CA07
2-5	H1	N1	CA07	CA07	CA07	CA07	CA07	CA07
2-6	H1	N1	CA07	CA07	CA07	CA07	CA07	CA07
2-7	H1	N1	CA07	CA07	CA07	CA07	CA07	CA07
2-8	H1	N1	CA07	CA07	CA07	CA07	CA07	CA07
2-9	H1	N1	CA07	CA07	CA07	CA07	CA07	CA07
2-10	H1	N1	CA07	CA07	CA07	CA07	CA07	CA07
2-11	H1	N1	CA07	CA07	CA07	CA07	CA07	CA07
2-12	H1	N1	CA07	CA07	CA07	CA07	CA07	CA07
2-13	H1	N1	CA07	CA07	CA07	CA07	CA07	CA07
2-14	H1	N1	CA07	CA07	CA07	CA07	CA07	CA07
2-15	H1	N1	CA07	CA07	CA07	CA07	CA07	CA07
2-16	H1	N1	CA07	CA07	CA07	CA07	CA07	CA07
3-1	H1	N1	CA07	CA07	CA07	CA07	CA07	CA07
3-2	H1	N1	CA07	CA07	CA07	CA07	CA07	CA07
3-3	H1	N1	CA07	CA07	CA07	CA07	CA07	CA07
3-4	H1	N1	CA07	CA07	CA07	CA07	CA07	CA07
3-5	H1	N1	CA07	CA07	CA07	CA07	CA07	CA07
3-6	WEAK	FAIL	FAIL	WEAK	FAIL	FAIL	FAIL	CA07
3-7	H1	N1	CA07	CA07	CA07	CA07	CA07	CA07
3-8	H1	N1	CA07	CA07	CA07	CA07	CA07	CA07
3-9	H1	N1	CA07	CA07	CA07	CA07	CA07	CA07
3-10	H1	N1	CA07	CA07	CA07	CA07	CA07	CA07
3-11	WEAK	FAIL	FAIL	FAIL	FAIL	FAIL	FAIL	FAIL
3-12	H1	N1	CA07	CA07	CA07	CA07	CA07	CA07
3-13	H1	N1	CA07	CA07	CA07	CA07	CA07	CA07
3-14	H1	N1	CA07	CA07	CA07	CA07	CA07	CA07
3-15	H1	N1	CA07	CA07	CA07	CA07	CA07	CA07
3-16	H1	N1	CA07	CA07	CA07	CA07	CA07	CA07
